# Wearable sensors for prediction of intraamniotic infection in women with preterm premature rupture of membranes: a prospective proof of principle study

**DOI:** 10.1007/s00404-022-06753-4

**Published:** 2022-09-13

**Authors:** Romana Brun, Julia Girsberger, Martina Rothenbühler, Catrin Argyle, Juliane Hutmacher, Christian Haslinger, Brigitte Leeners

**Affiliations:** 1 Department of Obstetrics, University Hospital Zurich, Frauenklinikstrasse 10, 8091 Zurich, Switzerland; 2 Department of Reproductive Endocrinology, University Hospital Zurich, Zurich, Switzerland; 3 University of Zurich, Zurich, Switzerland; 4 Ava AG, Zurich, Switzerland; 5 Department of Gynecology and Obstetrics, Cantonal Hospital Frauenfeld, Frauenfeld, Switzerland

**Keywords:** Wearable sensor, Preterm premature rupture of membranes, Intraamniotic infection, Photoplethysmography

## Abstract

**Purpose:**

To evaluate the use of wearable sensors for prediction of intraamniotic infection in pregnant women with PPROM.

**Materials and methods:**

In a prospective proof of principle study, we included 50 patients diagnosed with PPROM at the University Hospital Zurich between November 2017 and May 2020. Patients were instructed to wear a bracelet during the night, which measures physiological parameters including wrist skin temperature, heart rate, heart rate variability, and breathing rate. A two-way repeated measures ANOVA was performed to evaluate the difference over time of both the wearable device measured parameters and standard clinical monitoring values, such as body temperature, pulse, leucocytes, and C-reactive protein, between women with and without intraamniotic infection.

**Results:**

Altogether, 23 patients (46%) were diagnosed with intraamniotic infection. Regarding the physiological parameters measured with the bracelet, we observed a significant difference in breathing rate (19 vs 16 per min, *P* < .01) and heart rate (72 vs 67 beats per min, *P* = .03) in women with intraamniotic infection compared to those without during the 3 days prior to birth.

In parallel to these changes standard clinical monitoring values were significantly different in the intraamniotic infection group compared to women without infection in the 3 days preceding birth.

**Conclusion:**

Our results suggest that wearable sensors are a promising, noninvasive, patient friendly approach to support the early detection of intraamniotic infection in women with PPROM. However, confirmation of our findings in larger studies is required before implementing this technique in standard clinical management.

**Supplementary Information:**

The online version contains supplementary material available at 10.1007/s00404-022-06753-4.

## What does this study add to the clinical work

**Table:** 

Early detection of intraamniotic infection in women with PPROM is an unsolved problem in obstetrics. In this study including 50 women with PPROM, wearable sensors are shown to be a promising tool to detect the presence of intraamniotic infection.	

## Introduction

Preterm premature rupture of membranes (PPROM), defined as rupture of the membranes before 37 weeks of gestation, remains a significant obstetric problem affecting 3–4% of all pregnancies [1]. Several risk factors, such as ascending infections, cigarette smoking, and multiple pregnancy, seem to increase the likelihood of PPROM [1]. The management of women with PPROM (expectant versus delivery) differs depending on the gestational age and the clinical situation. PPROM can lead to intraamniotic infection with consequent preterm delivery. Despite intensive research until today, 40–50% of all preterm births are related to PPROM. To predict which women will develop an intraamniotic infection, needing to be delivered prematurely, and which will not, remains one of the major unsolved problems in obstetrics.

Acute chorioamnionitis, the histopathological counterpart of the clinical diagnosis of intraamniotic infection is diagnosed in 50–60% of women with PPROM [2, 3]. As chorioamnionitis poses a danger to the fetus by promoting early-onset sepsis and neonatal morbidities, such as cystic periventricular leukomalacia and cerebral palsy, its early and accurate diagnosis and treatment are indispensable [4]. Moreover, the mother is also at risk of developing sepsis, and may request admission to an intensive care unit [5]. Currently, serial measurements of C-reactive protein and leucocytes serve to monitor the development of infection in women with PPROM.

Since invasive diagnostic procedures to detect infection (such as serial blood samples, or even amniocentesis) are inconvenient for women, come with risks, and are costly, noninvasive methods represent a desirable alternative [6, 7]. In addition, clinical measurements can only be performed at isolated timepoints, while modern wearables permit continuous measurements over longer time periods.

Wearable sensors are enjoying broad popularity for tracking physical activity (fitness trackers and smartwatches) and are increasingly applied in the medical field. Recent advances in technology have made a wide range of innovative wearable sensor devices available, which enable the measurement and collection of health-related data [8]. Previous studies have already examined the use of wearable technology in several therapeutic areas [9], including cardiology [10] neurodegenerative diseases [11], oncology [12] and intensive care medicine [13]. This technology has also been shown to be an accurate tool to measure pulse rate (PR) in healthy individuals during rest and sleep [14–16]. Moreover, photoplethysmographic (PPG) spectral analysis has been evaluated for the noninvasive assessment of peripheral vascular regulation in sepsis patients, demonstrating potential implications for monitoring sepsis progression [17].

Through continuous or frequent monitoring of physiological parameters, patterns or changes in a patient’s health status can be detected and clinical deterioration may even be registered earlier than through standard vital sign monitoring [18–20]. Several studies conducted during the global COVID-19 pandemic have shown the ample potential of wearable devices for the detection of early periods of the infection [21–23].

Smart technology also plays a major role in the field of women’s health, ranging from improving health awareness for chronic diseases to supporting mental or maternal health [24, 25]. Wearable devices, such as smart bracelets, are promising tools for maternity care [26, 27] and monitoring of physiological parameters may allow early detection of pregnancy complications in more advanced ways than ever before [24].

Despite the clinical relevance of early infection detection, encouraging results from previous studies as well as successful use of wearables in pregnant women for other indications [24, 27–30], to our knowledge, no study on the usability of wearable sensors for prediction of intraamniotic infection has been performed yet. We therefore evaluate the detection of infection in women with PPROM using non-invasive parameters measured by a wearable device within a prospective proof of principle study.

## Materials and methods

### Study design

In a prospective proof of principle study, we included 50 patients diagnosed with PPROM at the University Hospital Zurich, a tertiary care perinatal center, between November 2017 and May 2020.

### Participants and consent

Participants included pregnant women aged 18 or more with PPROM diagnosis (defined as preterm premature rupture of membranes occurring before 37 weeks of gestation). Excluded were women < 18 years and women without PPROM or absence of informed consent.

### Procedures

Patients received the wearable sensor bracelet Ava (Ava AG, Zürich, Switzerland) and were instructed by a clinical nurse to wear the wearable device on the dorsal side of their wrist nightly whilst sleeping. As daily activities influence data quality, women were instructed to wear the device during the night only, when they are confronted with fewer confounding factors and measurements can be realized under more standardized conditions.

The electronic wearable automatically saved physiological information every 10 s throughout the night. Participants were shown how to synchronize the device with the complementary app on their smartphone and were instructed to do so each morning upon waking. In case of technical problems, the clinical nurse was informed and visited the woman on the prenatal ward to provide support.

Prospective data collection was performed by dedicated research personnel using patient data from the general and obstetrics-specific clinical information systems.

Data on the following obstetric and neonatal parameters were collected: maternal age, pre-pregnancy BMI, ethnicity, parity, twin pregnancies, gestational age at PPROM, gestational age at delivery, cervical length at admission (normal cervical length is considered to be > 30 mm [31]), administration of antibiotics, duration of antibiotic administration, time between PPROM and delivery, induction of labor, mode of delivery, clinical suspicion of intraamniotic infection, histopathological diagnosis of chorioamnionitis, birth weight, umbilical cord pH, 5 min Apgar score, 10 min Apgar score, transfer to neonatal intensive care unit, and administration of antibiotics to neonate. Furthermore, clinical parameters such as pulse rate, body temperature, color of the amniotic fluid, baseline of the cardiotocogram, as well as laboratory tests such as CRP and leucocytes, were collected.

The diagnosis of intraamniotic infection was clinically suspected if the pregnant woman presented with fever and one or more of the following parameters: maternal tachycardia, fetal tachycardia, elevated serum infection parameters (CRP or leucocytes) or purulent fluid from the cervical os. If the body temperature is normal, it is measured once a day in ear. If the temperature is > 37.5 °C, repeat measurements will be done. Fever is defined as a body temperature > 38 °C. Maternal tachycardia is defined as heart beat frequency of > 100 bpm. Fetal tachycardia is defined as heart beat frequency of > 160 bpm for more than 10 min. Elevated serum levels of CRP are > 5 mg/l. For non-pregnant women, the cut-off of our laboratory for leucocytes is 9.6G/l. As during pregnancy, leukocyte levels are physiologically increased, levels over 15 G/l are considered as leukocytosis [32]. In our institution, in case of borderline leukocyte values, we rather consider the dynamics than the absolute numbers. A decision on premature delivery was taken when an intraamniotic infection according to the above-mentioned criteria was suspected. Final diagnosis was based on confirmation of chorioamnionitis in histopathological evaluation after delivery. However if the clinical diagnosis of intraamniotic infection was made, we do not change the diagnosis afterwards if the criteria for the histopathological diagnosis of chorioamnionitis were not fulfilled. This is because we think that there might be cases where the clinical diagnosis and the clinical symptoms of intraamniotic infection present earlier (e.g. depending on the maternal immune system or after antibiotic treatment). In these cases, the delivery might have been that prompt that the infection has not already attacked the placenta.

Our pathologists comply their histopathological diagnosis according to the Amsterdam definitions (Amsterdam Placental Workshop Group Consensus Statement) [33]. In this statement it is also mentioned that there was an agreement to emphasize that histologic chorioamnionitis may not be equivalent to clinical chorioamnionitis.

The protocol for the medical care of women with PPROM at our prenatal ward is the following: daily control of maternal pulse, body temperature, the color of amniotic liquid and CTG, blood samples (CRP and leucocytes) twice a day for the first 3 days, and in case of normal values once a day for the following 7 days, and subsequently once a week until delivery. A vaginal smear is performed at admission. Every woman receives prophylactic antibiotic treatment for 7 days (macrolide antibiotics such as Clarithromycin or Erythromycin; or Penicillin in presence of group B streptococci in the vagina). If the vaginal smear is positive for bacteria, the antibiotic treatment is adapted according to the antibiogram.

As PPROM poses a risk for imminent delivery, lung maturation is recommended if PPROM occurs before 34 weeks of gestation. Therefore, tocolytic therapy is often used to stop any contractions to prolong the pregnancy during the time of lung maturation (corticosteroid administration twice every 24 h) i.e. for 48 h or even longer in some cases. We use several tocolytic agents: nifedipine (calcium channel blocker), hexoprenaline (betamimetic drug) or atosiban (oxytocin receptor antagonist).

In the absence of suspected intraamniotic infection, the pregnancy can be prolonged until 37 weeks of gestation. At that point, an induction of labor is discussed with the pregnant woman, since after 37 weeks of gestation, the risk of adverse outcomes (especially infection risk in case of waiting) outweighs the risk of preterm delivery.

### Materials

Women wore the Ava bracelet (Ava AG, Zürich, Switzerland) as a wearable device to measure the physiological parameters used for the present analysis, i.e. wrist skin temperature (WST), pulse rate (PR), heart rate variability (HRV), and breathing rate. These parameters are measured through a PPG sensor, temperature sensors and a three-axis accelerometer [14]. PPG is a noninvasive, cost-effective optical technique, which enables the monitoring of HRV, PR, skin perfusion and breathing rate by tracking alterations of blood volume via changes in absorption and reflection of light from a light-emitting diode (LED) and photodiodes [18, 34]. This technique uses red and infrared light to analyze the oxygen content of blood, but also produces a waveform that represents instantaneous changes in blood volume within a body area, and therefore may carry important information about control, performance and compensatory changes occurring within the cardiovascular system [17].

### Statistical analyses

The primary outcome was the difference in wearable device measured physiological parameters and clinical measurements between women with and without intraamniotic infection during the 3 days prior to birth. We chose the 3 days prior to birth because we expected this time to provide physiological evidence of an infection, without the effect of eventual contraction pain on the day of birth.

Baseline and clinical characteristics are presented as mean (SD), median (interquartile range [IQR]) or counts (percentage) as appropriate.

A total of 50 women were included in the baseline analysis (baseline and clinical outcomes). Of these 50 women, 44 collected and synchronized the wearable device measured parameters and were included in the analysis of the primary outcome.

We compared the parameters measured by the wearable device, such as wrist skin temperature, heart rate, heart rate variability, and breathing rate, according to presence or absence of intraamniotic infection. Furthermore, we compared the standard clinical measurements to diagnose intraamniotic infection, i.e., leukocytes, CRP, core temperature and pulse in the two groups. Differences between groups are illustrated using Boxplots and corresponding means, confidence intervals were determined by woman and group separately. A two-way repeated measures ANOVA was performed to evaluate the effect of intraamniotic infection on these measurements over time. Two-sided *p* values with an alpha level of 0.05 were considered statistically significant. All statistical analyses were performed using R version 3.6.0.

The HRV used in this analysis is the filtered median Standard Deviation of the NN intervals (SDNN), the “gold standard” for medical stratification of cardiac risk [35]. Abnormal beats were removed and the median value of the standard deviation of the interbeat intervals of normal sinus beats was estimated per night and woman.

### Ethical approval

All researches were performed in accordance with the Declaration of Helsinki. The clinical protocol was reviewed and approved by the Cantonal Ethics Committee Zurich, Switzerland (BASEC -2016–02,241). Informed consent was obtained from all the study participants before their study involvement.

## Results

### Population characteristics

**Fig. 1:**
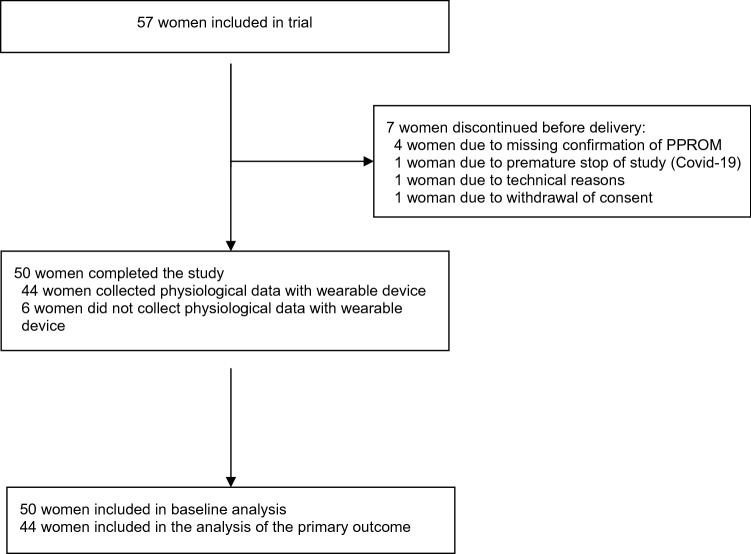
Flowchart

From the initially recruited 57 women, we had to exclude seven patients (see Fig. 1). In four of them, the initial diagnosis of PPROM could not be confirmed. One woman failed to synchronize her bracelet with the cellphone app and one woman withdrew her consent. As we had to terminate the study recruitment ahead of schedule in May 2020 because of the COVID-19 pandemic, the last recruited patient could not conduct measurements in agreement with the study design. The final sample included 50 women’s data, of whom 44 had collected physiological data with the wearable device and were included in the analysis of the primary outcome.

The baseline characteristics of the 50 patients who participated in the study are summarized in Table 1. On average, participants synchronized their bracelet for 9.5 days (IQR 3.25–22.75) between PPROM and labor. Altogether, all 50 (100%) participating women received antibiotic treatment with an average duration of 6.22 days (SD 2.61).

**Table 1:** Baseline characteristics women

Number of participants	50
Age (years), mean (SD)	33.8 (5.8)
BMI (kg/m^2^), mean (SD)	24.3 (4.9)
Ethnicity, *n* (%)	
Afro-Caribbean	1 (2)
Asian	5 (10)
Mediterranean	6 (12)
Caucasian	38 (76)
Nulliparity, *n* (%)	36 (72)
Twins, *n* (%)	11 (22)
Dichorial-diamniotic	8 (16)
Monochorial-diamniotic	3 (6)
Gestational age at PPROM (weeks, days), median (IQR)	30.7 weeks (223 days, IQR 197–234)
Gestational age at delivery (weeks, days), median (IQR)	32.9 weeks (236 days, IQR 220–247)
Cervical length at admission (mm), mean (SD)	28 (13)
Antenatal steroid prophylaxis	38 (76%)
Administration of antibiotics,* n* (%)	50 (100)
Duration of antibiotic administration (days), mean (SD)	6.2 (2.6)

Data are shown as counts (percentage), mean (standard deviation) or median (IQR)

*IQ* interquartile range. *SD* standard deviation

### Outcomes

Maternal and neonatal outcome parameters are shown in Table 2. The median time between PPROM and delivery was 9.5 days (IQR 3.3–22.8). Labor had to be induced in 7 (14%) women. Of the 50 participants, 19 (38%) gave birth through vaginal delivery, while 31 (62%) had a cesarean section. In total, 23 (46%) women were diagnosed with intraamniotic infection, whereas the histopathological diagnosis of chorioamnionitis was made in 20 (40%) women.

**Table 2:** Maternal and neonatal outcomes

Women (*n* = 50)	
Time between PPROM and labor (days), median (IQR)	9.5 (IQR 3.25−22.75)
Induction of labor, *n* (%)	7 (14)
Mode of delivery, *n* (%)	
Vaginal birth	18 (36)
Operative vaginal birth	1 (2)
Planned cesarean section	1 (2)
Unplanned cesarean section	30 (60)
Delivery ≥ 37 weeks of gestation, *n* (%)	7 (14)
Premature delivery (< 37 weeks of gestation), *n* (%)	43 (86)
32−37 weeks of gestation, *n* (%)	29 (58)
28−32 weeks of gestation, *n* (%)	9 (18)
< 28 weeks of gestation, *n* (%)	5 (10)
Clinically suspected intraamniotic infection, *n* (%)	23 (46)
Histopathological diagnosis of chorioamnionitis, *n* (%)	20 (40)
Neonates (*n* = 60)	
Birth weight (grams), mean (SD)	1929.75 (701.11)
Arterial umbilical cord pH, mean (SD)	7.29 (0.08)
5 min Apgar < 7, *n* (%)	8 (13.3)
10 min Apgar < 7, *n* (%)	1 (1.7)
Transfer to a neonatal intensive care unit, *n* (%)	43 (71.7)
Administration of antibiotics to neonate, *n* (%)	20 (33.3)
Early onset neonatal sepsis	1 (1.7)

Data are shown in counts (percentage), mean (standard deviation) or median (IQR)

*IQR*: interquartile range, *SD*: standard deviation

There was a statistically significant correlation between intraamniotic infection and chorioamnionitis (*p* < 0.001), Table 3. Altogether, 43 (86%) women had a premature delivery. The mean birth weight of the babies was 1929 g (SD 701.11). A total of 43 babies had to be transferred to a neonatal care unit (71.7%) and 20 of them had to be treated with antibiotics (33.3%).

**Table 3:** Correlation between intraamniotic infection and chorioamnionitis

	No chorioamnionitis	Chorioamnionitis
No intraamniotic infection	27	0
Intraamniotic infection	3	20

*P* < 0.001

### Primary outcome

**Fig. 2:**
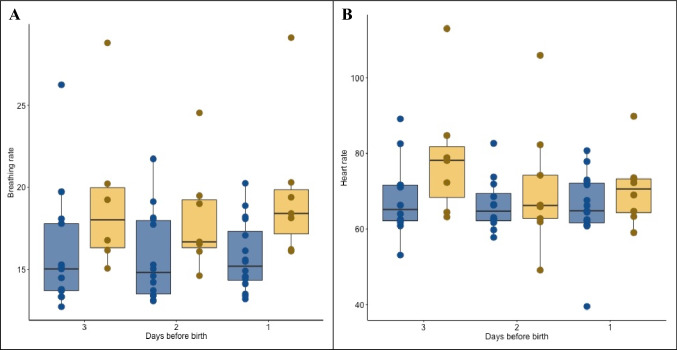
Breathing and heart rate in the 3 days before birth according to intraamniotic infection. Wearable sensor measured breathing rate (Panel **A**) and heart rate (Panel **B**) in the 3 days prior to birth. Blue boxplots visualize measurements from patients without intraamniotic infection, yellow boxplots visualize measurements from patients with intraamniotic infections. Every point represents the measurement of one patient

Regarding the investigated physiological bracelet parameters, we observed a significant difference in breathing rate (19 versus 16 per min, *P* < 0.001) and heart rate (72 versus 67 beats per min, *P* = 0.03) in women with intraamniotic infection (n = 19, 43.2%) compared to those without infection (*n* = 25, 56.8%) during the 3 days prior to birth. Significant findings from the two-way repeated measures ANOVA are presented in Table 4. In addition, significant differences in breathing rate and heart rate captured by the wearable device during the 3 days prior to birth are illustrated in Fig. 2.

**Table 4:** Difference of wearable device measured physiological parameters and clinical measurements between women with and without clinically suspected intraamniotic infection in the 3 days prior to delivery (*n* = 44)

	Wearable device measured parameters *(n* = 44)				Clinical measurements (*n* = 50)			
	Breathing rate (n/min)	Heart rate (beat/min)	Wrist skin temperature (C°)	Heart rate variability (ratio)	CRP (mg/l)	Leucocytes (G/l)	Body temperature (C°)	Pulse (beat/min)
Overall	17.2 (15.9 − 18.4)	69.1 (65.4−72.8)	34.8 (34.6−35.1)	55.8 (50.9−60.7)	7.9 (5.3−10.5)	11.6 (10.6−12.5)	36.9 (36.9 to 37.0)	91.1 (88.1 to 94.1)
Women with intraamniotic infection (*n* = 19)	19.1(16.7−21.4)	71.8 (64.7−79.0)	34.8 (34.4−35.2)	53.2 (45.5−61.0)	11.3 (6.3−16.3)	12.6 (10.9−14.3)	37.0 (36.9 to 37.1)	94.9 (91.0 to 98.8)
Women without intraamniotic infection (*n* = 25)	15.9 (14.8−17.0)	67.2 (62.9−71.5)	34.9 (34.6−35.2)	57.5 (50.6−64.4)	5.0 (3.2−6.8)	10.6 (9.7−11.5)	36.9 (36.7 to 37.0)	87.9 (83.6 to 92.2)
*P* value for difference between groups	0.001	0.03	0.87	0.12	0.02	0.04	0.03	0.02

As expected, the standard clinical monitoring values, such as C-reactive protein (CRP) and leucocytes, were also significantly different in the intraamniotic infection group compared to women without infection during the 3 days preceding birth.

The data presented in Table 3 represent the mean and corresponding 95% CI of point estimates of the clinical measurements and of percentiles throughout one day per woman of the wearable device measurements. For the heart rate, we use the 10th percentile, for the temperature, the 99th percentile and for the breathing rate and the heart rate variability we used the median. Previous work by the manufacturer has shown that the 10th percentile of the heart rate is more robust to outliers, and that the 99th percentile of the temperature is closest to the body temperature.

Wearable sensor measured breathing rate (Panel A) and heart rate (Panel B) in the 3 days prior to birth. Blue boxplots visualize measurements from patients without intraamniotic infection, yellow boxplots visualize measurements from patients with intraamniotic infections. Every point represents the measurement of one patient.

For the other measured physiological parameters with the wearable device (wrist skin temperature, heart rate variability, and skin perfusion), no statistical significance could be determined in the intraamniotic infection group compared to women without infection in the 3 days preceding birth.

Additional information regarding the association of these physiological parameters and the intraamniotic infection is given in the supplementary information section by displaying the operating characteristic (ROC) curves and the corresponding area under the curve (AUC) measures.

## Discussion

This prospective proof of principle study showed a significant difference in wearable device measured breathing rate and heart rate when comparing women hospitalized for PPROM with intraamniotic infection to those without in the 3 days preceding delivery. Our results show a significant difference in wearable device measured breathing rate (19 versus 16 per min,* p* < 0.01) and heart rate (72 versus 67 beats per min, *p* = 0.03) during the 3 days prior to birth in women with intraamniotic infection compared to those without infection.

Infection changes different corporal functions, which allow its diagnosis, and which can be monitored by electronic wearables. It can develop from a local entry of bacteria into the body, in this case from the vagina through the opened membranes into the amniotic liquid [36]. This infection may affect the total body and eventually cause sepsis, a life-threatening situation and a medical emergency [37], which is the reason why amniotic infection needs to be detected and treated as early as possible. Also, such infection causes contractions and consequently premature birth, with all its known consequences of potential physical and mental handicaps [36]. In addition, babies should not be exposed to infection for a longer than necessary time as several studies show that premature infants with peripartal infections have poorer outcome compared to those without infection [38, 39]. Such situations can only be prevented with early detection of a developing infection. However, clinical development following PPROM, i.e., if, and especially when, infection occurs despite systematic antibiotic treatment, is highly individual and currently available parameters do not allow prediction of the clinical course. Therefore, early diagnosis is mandatory to design a safe treatment for mother and child, with the best possible long-term outcome.

Typically, with the onset of infection, heart rate increases, body temperature rises, shortness of breath leads to increased breathing rate, and the heart rate variability changes its pattern and decreases [40, 41]. According to our findings, breathing rate and heart rate measured by an electronic wearable may indeed capture these physiological changes related to infection.

Our data also show a tendency towards a lower heart rate variability in women with intraamniotic infection compared to those without infection. Heart rate variability, i.e., the variation in beat-to-beat intervals of the heart, reflects the ability of the system to react to stressors [42] and decreases in stress situations. An infection in participants with PPROM can be considered as a stressor. Continuous HRV monitoring has been shown to reduce the gap between the onset of sepsis and its clinical recognition, allowing clinicians to direct early intervention efforts in sepsis treatment [43, 44]. According to our results, the continuous measurements of the heart rate and its variability therefore also have promising potential for the prediction of early intraamniotic infection when monitoring women with PPROM. In contrast to the serial timepoint measurements of parameters currently used to monitor women after PPROM and diagnose amniotic infection, wearable devices such as the one used in our study allow continuous longitudinal measurements, so that changes can be captured directly at the moment of their occurrence. Also, their noninvasive nature is more convenient than, for example, blood sampling. A further advantage of wearable devices is that in contrast to blood samples they also offer the possibility of home monitoring after hospital discharge.

Standard clinical monitoring values, such as C-reactive protein (CRP) and leukocytes, were significantly different in the intraamniotic infection group compared to women without infection in the three days preceding delivery, in agreement with the criteria leading to suspicion of intraamniotic infection. Also, the core body temperature measured as a clinical parameter showed a significant difference in women with compared to those without intraamniotic infection.

To our astonishment, we did not find any augmentation in wrist temperature, although temperature changes in relation to the menstrual cycle can be reliably measured with the bracelet [45, 46]. Since body temperature varies during the 24 h of the day, this might explain why the temperature measured with the wearable device worn only nightly did not show any significant changes in relation to intraamniotic infection. Future studies including a higher frequency of core body temperature measurements would allow to better understand such associations. However, as it is the early changes of infection we are aiming to detect, our findings do support the advantages of using a wearable for detection of intraamniotic infection.

The heart rate significantly differed 3 days before the diagnosis of intraamniotic infection, however, the day before birth we can only see a non-significant difference of the median heart rate (Wilcoxon *U*-Test *p* = 0.33). As this study was designed as a proof of principle study being the first study regarding these parameters in women with PPROM, we cannot compare the measured values to other published data. one possible explanation could be confounding factors such as tocolytic agents or other co-medication influencing the heart rate during this timeframe. Another reason could be the small sample size allowing no sub-analyses for individual parameters.

To our best knowledge, this is the first study to analyze this noninvasive approach to intraamniotic infection detection.

A strength of our study is the strong correlation between the clinical diagnosis of intraamniotic infection and the histopathological diagnosis of chorioamnionitis, confirming the reliability of our diagnosis.

As the clinical development in our patients varied considerably, our study group is rather inhomogeneous with regard to gestational age at the time of PPROM, the time interval between admission and delivery, medication received during hospitalization, etc. Although our findings clearly demonstrate that physiological changes occurring in association with infection can be captured by a nightly worn electronic device, the sample size does not allow any sub-analyses for individual developments. Therefore, larger studies are needed in the future for more differentiated analyses, also with regard to the timeline of changes in individual parameters. Such studies would also allow to adjust for confounders, for example tocolytic agents, which influence the heart rate and heart rate variability and might have influenced findings. However, side effects of tocolytics do not conceal an ongoing infection.

Moreover, the antenatal steroid prophylaxis is able to influence some measured parameters (leukocytes or temperature). 76% of all patients received antenatal steroid prophylaxis. In our department, we administer antenatal steroid prophylaxis until 34 weeks of gestation. In the group of women included before 34 weeks of gestation, only one woman did not receive antenatal steroid prophylaxis. It cannot be ruled out that antenatal steroid prophylaxis influence some measured parameters, however, if there should be a bias it would be a bias for almost all women of the study.

Although the amount of available synchronized data across participants varied strongly, irrespective of the number of days spent in hospital information on the parameters requested was always available either for the full hospitalization time prior to delivery or for at least three days prior to delivery. Consequently, such measurements could help to improve early detection, allowing to adjust clinical procedures such as the timing of delivery, and to prevent unfavorable effects on neonatal outcome. Timing in intraamniotic infection is important because premature babies should not be exposed to an infection for a longer time than necessary as several studies show that premature infants with peripartal infections have poorer outcome compared to those without infection [38, 39].

Medical induction of labor due to PPROM, which in our clinic is performed at 37 weeks of gestation in the absence of infection, could be interpreted as a bias because the “natural” course of pregnancy (and potentially infection) is intercepted prematurely; however, the induction rate of our study population was only 14%.

## Conclusion

This proof of principle study demonstrated the potential of a wearable device to noninvasively measure physiological parameters related to the development of intraamniotic infection after PPROM, consequently allowing its early detection.

Larger studies will help to identify the predictive capabilities of these parameters for different clinical outcomes in PPROM patients, and to understand whether and how noninvasively measured parameters could support future clinical decision making.

## Supplementary Information

Below is the link to the electronic supplementary material.

Supplementary file1 (DOCX 100 KB)
