# Supplementary material for: Wearable sensors for prediction of intraamniotic infection in women with preterm premature rupture of membranes: a prospective proof of principle study
Source: Arch Gynecol Obstet. 2022 Sep 13;308(5):1447–56. doi: 10.1007/s00404-022-06753-4 (PMC9469066; doi:10.1007/s00404-022-06753-4)
Supplement: Supplementary file 1 — Supplementary file1 (DOCX 100 KB) [file 404_2022_6753_MOESM1_ESM.docx]

**Supplementary Information**

Table S1. Baseline characteristics women divided into the subgroups intraamniotic infection and no intraamniotic infection

| Women (n=50) | |  |  |
| --- | --- | --- | --- |
|  | | With intraamniotic infection n= 23 (46%) | Without intraamniotic infection n =27 (54%) |
| Age (years), mean (SD) | | 33.6 (6.1) | 34 (5.6) |
| BMI (kg/m^2^), mean (SD) | | 24.5 (5.48) | 24.2 (4.4) |
| Ethnicity, n (%) | |  |  |
|  | Afro-Caribbean | 0 (0) | 1 (3.7) |
|  | Asian | 4 (17.4) | 1 (3.7) |
|  | Mediterranean | 4 (17.4) | 2 (7.4) |
|  | Caucasian | 15 (65.2) | 23 (85.2) |
| Nulliparity, n (%) | | 15 (65.2) | 21 (77.8) |
| Twins, n (%) | | 4 (17.4) | 7 (26) |
|  | Dichorial-Diamniotic | 3 (75) | 5 (71.4) |
|  | Monochorial-Diamniotic | 1 (25) | 2 (28.6) |
| Gestational age at PPROM (weeks, days), median (IQR) | | 31.4 weeks (222 days, IQR 194-229) | 32.4 weeks (227 days, IQR 204-240) |
| Gestational age at delivery (weeks, days), median (IQR) | | 32.4 weeks (227 days, IQR 202-236) | 34.7 weeks (243 days, IQR 236-256) |
| Cervical length at admission (mm), mean (SD) | | 27 (17) | 28 (8.8) |
| Administration of antibiotics, n (%) | | 23 (100) | 27 (100) |
| Duration of antibiotic administration (days), mean (SD) | | 6 (2.3) | 6.4 (2.9) |

Table S2. Maternal and neonatal outcomes divided into in the subgroups intraamniotic infection and no intraamniotic infection

| Women (n=50) | | |  |
| --- | --- | --- | --- |
|  | | With intraamniotic infection n= 23 (46%) | Without intraamniotic infection n=27 (54%) |
|  | | |  |
| Time between PPROM and labor (days), median (IQR) | | 6.0 (IQR 3.5 – 11.5) | 15 (IQR 4.5-35) |
| Induction of labor, n (%) | | 2 (8.7) | 5 (18.5) |
| Mode of delivery, n (%) | |  |  |
|  | Vaginal birth | 7 (30.4) | 11 (40.7) |
|  | Operative vaginal birth | 0 (0) | 1 (3.7) |
|  | Planned cesarean section | 0 (0) | 1(3.7) |
|  | Unplanned cesarean section | 16 (69.6) | 14 (51.9) |
| Delivery ≥ 37 weeks of gestation, n (%) | | 1 (4.3) | 6 (22.2) |
| Premature delivery (< 37 weeks of gestation), n (%) | | 22 (95.7) | 21 (77.8) |
|  | 32 -37 weeks of gestation, n (%) | 12 (52.2) | 17 (63) |
|  | 28- 32 weeks of gestation, n (%) | 7 (30.4) | 2 (7.4) |
|  | < 28 weeks of gestation, n (%) | 3 (13) | 2 (7.4) |

Table S 3: Median values of diagnostic criteria for intraamniotic infection in women with or without intraamniotic infection at the day of delivery

|  | Women with intraamniotic infection | No intraamniotic infection | p-value for difference |
| --- | --- | --- | --- |
| Fetal heart beat (in beats/minutes) | 150 (140 to 160) | 140 (135 to 140) | <0.001 |
| Maternal pulse rate | 104 (84.5 to 107) | 86 (80 to 91) | 0.015 |
| Body temperature | 37.5 (37.2 to 37.6) | 36.8 (36.7 to 37.05) | <0.001 |

Data are shown as median (IQR), IQR: interquartile range

Table S 4: AUC

|  | **AUC** |
| --- | --- |
| Leucocytes | 0.6319 |
| Body temperature | 0.6472 |
| CRP | 0.6473 |
| Pulse | 0.6522 |
| Breathing rate | 0.7018 |
| Heart rate | 0.6491 |

Figure S1: ROC


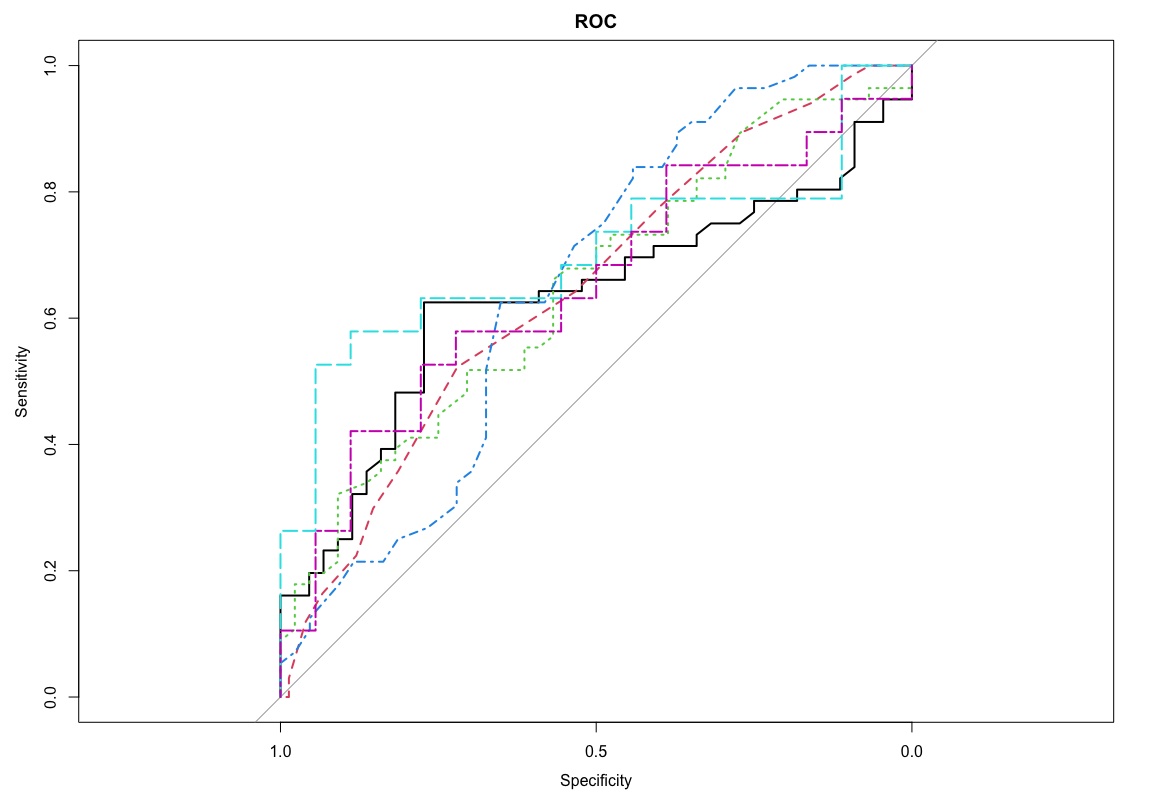


Legend: Black solid line: leucocytes, Dashed red line: body temperature, Short dashed green line: CRP, Normal-short dashed blue line: pulse, Long dashed turquoise line: breathing rate

Purple line: heart rate
